# Ecological assessment of coal mine and metal mine drainage in South Korea using *Daphnia magna* bioassay

**DOI:** 10.1186/s40064-015-1311-1

**Published:** 2015-09-17

**Authors:** Sang-Ho Lee, Injeong Kim, Kyoung-Woong Kim, Byung-Tae Lee

**Affiliations:** School of Environmental Science and Engineering, Gwangju Institute of Science and Technology, Gwangju, Republic of Korea

**Keywords:** Acid mine drainage (AMD), *Daphnia magna*, Whole effluent toxicity (WET), Toxicity identification evaluation (TIE), Cumulative criterion unit (CCU)

## Abstract

In order to assess the ecological effect of acid mine drainage, metal mine (Dalsung) and coal mine (Samtan) drainage in South Korea were collected. The each mine drainage then investigated by whole effluent toxicity test (WET) and toxicity identification evaluation (TIE). WET results demonstrated that DS leachate and ST mine water is more toxic than other mine drainage due to the presence of cationic metals and acidic pH. TIE results revealed that the acidic pH and copper (Cu) could be the main toxicants in both mine drainage. The strong acidic pH (pH < 3.5) enhanced the metal toxicity by increase of metal activity and bioavailability. The toxicity of most mine drainage revealed that the positive correlation between metal concentration and toxicity unit (TU). The regression data between TU and sum of cumulative criterion unit (CCU) demonstrated the reasonable statistical significance (R = 0.89; p < 0.01), however the excessive iron concentration in mine drainage could be an inhibition factor to estimate the toxicity by the effect of amorphous iron precipitate.

## Background

Acid mine drainage (AMD) is acidic sulfur-rich wastewater created from mine areas by the oxidation of sulfidic minerals (Gazea et al. 1996; Nordstrom and Alpers 1999; Johnson and Hallberg 2005; Kalin et al. 2006). In order to assess the negative impacts of AMD, conventional chemical measurement such as pH, conductivity, and metal and anion concentrations have been applied, as they can be simply compared based on the concentration difference of the components (Gray 1997). However, such a simple measurement of chemical concentrations has limits for use in estimating the negative effects of AMD as there are numerous hazardous factors interacting, i.e., elements can react with each other and form non-hazardous precipitates; therefore, a chemical measurement cannot represent the overall negative effect (Banks et al. 1997; Lopes et al. 1999; Hui et al. 2005). Consequently, biological assessments have been used to overcome the problems of chemical assessments because they can be used to systematically assess the effects of contaminated water, including wastewater and AMD (Yim et al. 2006; Mishra et al. 2008).

Toxicity tests using *Daphnia magna* are considered an effective biological assessment of aquatic environments because of various advantages such as broad distribution, ease of cultivation in laboratory, short life cycle, high reproduction rate, and sensitivity to toxicants (Farré and Barceló 2003). Indeed, the US Environmental Protection Agency (USEPA) has standardized the assessment methodology for using *Daphnia magna*; they suggest the use of whole effluent toxicity test (WET) and toxicity identification evaluation (TIE) methods to estimate the biological assessment of contaminated water such as industrial wastewater and AMD (US Environmental Protection Agency 2002). The WET method is an integrated tool used to measure the toxicity of wastewater that is comprised of a diverse number of undefined toxicants. In contrast, the TIE method can be used to identify the main cause of toxicity, in which the procedures consist of the fractionation of wastewater by either physical and/or chemical manipulations. Both WET and TIE have been successfully applied to identify toxicants in industrial wastewater and environmental samples (Villamar et al. 2011; de Melo et al. 2013).

To date, previous studies on the assessment of AMD has mainly focused on its chemical analysis and characterization. AMD characterization should be accompanied with a biological assessment owing to its inherent characteristics such as acidic pH and high concentrations of sulfates and heavy metals. However, there was a few studies about the bioassay of AMD with chemical analysis. Therefore, the purpose of this study is to evaluate the negative effects of AMD through a biological assessment using *Daphnia magna*. For this task, a representative coal mine and metal mine in South Korea were geochemically characterized to address the water contaminants selected for a comparison of the effects of AMD characteristics. The toxicities of water samples were then compared by WET and TIE was applied to identify the main toxicant in each acid mine drainage.

## Methods

**Fig. 1:**
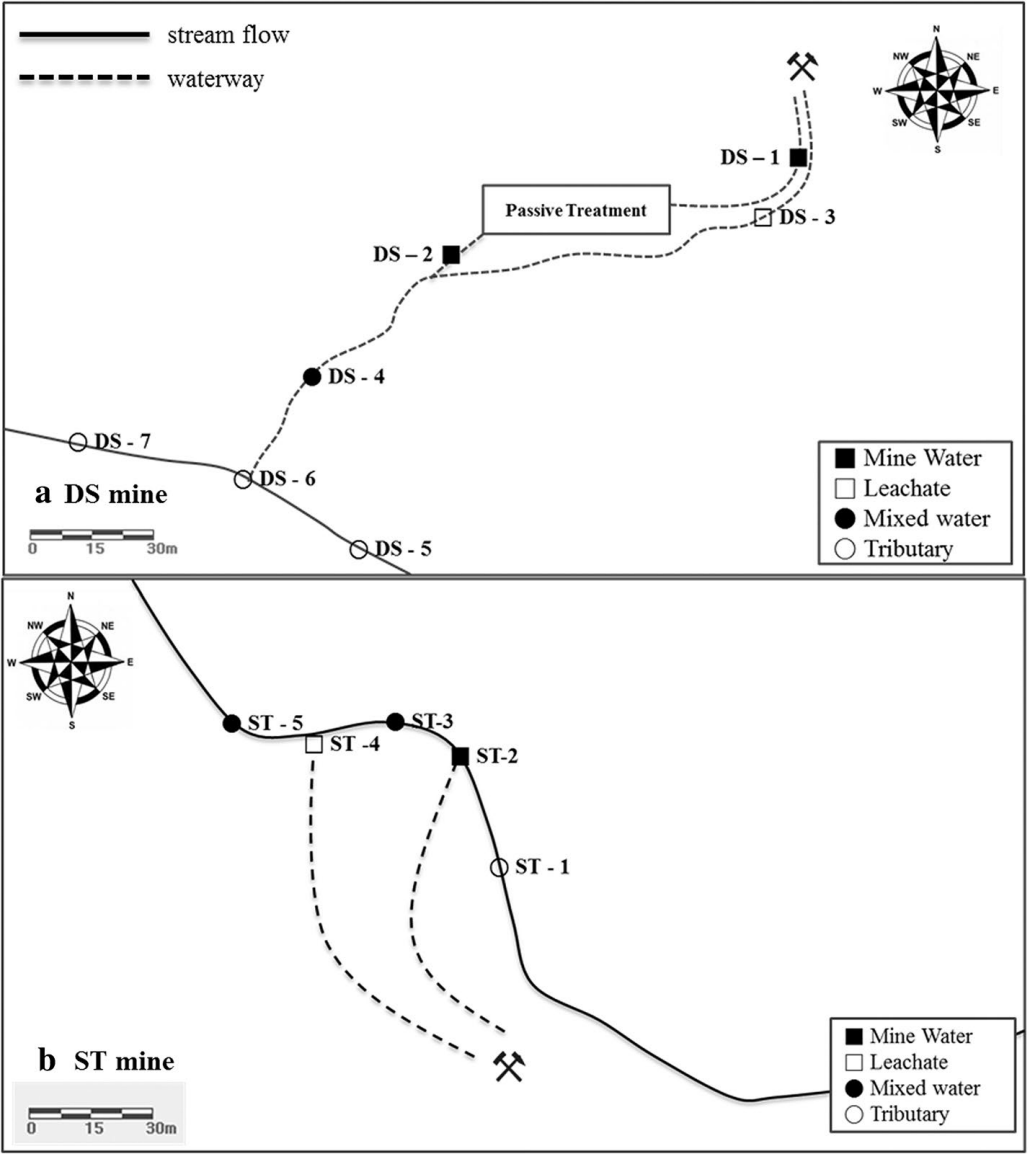
Sampling location of mine drainage in Dalsung Mine (DS **a**) and Samtan Mine (ST **b**)

Study sites were selected by the characteristics of mine drainage, with the Dalsung mine (DS) in Daegu and Samtan mine (ST) in Jeongseon ultimately being selected as representative study sites in this research. The study sites are illustrated in Fig. 1. DS is a copper and tungsten mine in South Korea. Though it was closed in 1994, mine drainage has been disch arged into adjacent streams. There is a passive remediation facility and the capacity is 2700 m^3^, and consists of a successively alkalinity producing system (SAPS) and a constructed wetland system. The flow rate of DS-1 is 27 ton/day and the mine drainage is actively being treated and discharged (DS-2; 26 ton/day). Furthermore, there is other mine drainage (DS-3; leachate) being discharged in addition to the passive treatment facility (flow rate of 19 ton/day). The treated water and leachate are mixed before entering the adjacent stream (DS-4), with a total flow rate of 46 ton/day. The mixed drainage is then discharged into an adjacent stream (DS-5) and diluted with 14,578 ton/day of upstream water in dry season. The mixed mine drainage subsequently creates a mixed zone (DS-6) with the stream and flows toward the downstream (DS-7). The Samtan mine (ST) is a representative coal mine in South Korea; the mine was closed in 2001. Since its closing, two types of mine drainage have been discharged into the adjacent stream without treatment. Currently, 3120 ton/day of leachate (ST-2) and 2083 ton/day of mine water (ST-4) are being discharged into an adjacent stream. Each mine drainage is mixed with 42,191 ton/day of adjacent stream (ST-1) water in dry season, which creates mixed zones along the stream flow (ST-3, ST-5).

On sites, the temperature, pH, dissolved oxygen (DO), oxidation reduction potential (ORP), total dissolved solids (TDS), salinity, and alkalinity were directly measured using a multi-meter (HI-9828, Multiparameter Water Quality Meter, HANNA, USA). In addition, the concentrations of total iron, ferrous, sulfate and sulfide ions were measured using a potable colorimeter (Drel 2400, HACH, USA). Total suspended solid was calculated by mass balance with 0.45 µm membrane filtration.

The samples were collected from the mine drainage from each mine and adjacent streams, from March to August in 2012. In the DS mine, the samples were collected on 28 March and 20 July; the samples from the ST mine were collected on 1 June and 26 August. The samples were divided two types such as chemical analysis and bioassay. For the chemical analysis, the samples were immediately filtered using a 0.45 μm cellulose ester membrane filter, then one sample was preserved using nitric acid (60 %; Dongwoo Chemical, Korea) to measure the cations (Al, As, Ca, Cd, Cu, Mn, Mg, Na, Zn) by inductively coupled plasma-optical emission spectroscopy (ICP-OES, 4300DV, Perkin Elmer, USA) in laboratory. For bioassay, the samples were collected in 4 L polyethylene containers, and stored below 4 °C to prevent microbial growth and metal precipitation.

Acute toxicity tests using *Daphnia magna* were performed using the WET procedure from the USEPA (U.S. Environmental Protection Agency 2007). Five neonates (<24 h old) of *Daphnia magna* were placed in 20 mL of the diluted mine water. In each test, several volumetrically diluted samples (e.g., 3.125, 6.25, 12.5, 25, 50, 100 %) were prepared using dilution of standard hard water which is referred USEPA method (USEPA, 2007), with four replicates conducted to measure the LC_50_ of the mine water. Culturing and exposure of *Daphnia magna* were conducted at 20 ± 2 °C in a photoperiod of 16 h light: 8 h dark condition, and the illumination was 900 ± 200 lux. The 24 h lethal concentrations (LC_50_) were then calculated by the probit and trimmed spearmanarber method (Hamilton et al. 1977). In order to compare the toxicity in acid mine drainage, LC_50_ values were transformed to toxicity units (TU). This transformation can be expressed as:

1 $${\text{TU}} = 100\, \% /{\text{LC}}_{ 50}$$

To identify the main toxicants of the acid mine drainage, a toxicity identification evaluation (TIE) procedure was conducted after the toxicity in the samples was determined. The TIE procedure consists of three steps; the Phase I procedure includes several treatments on the samples to determine the main toxic effect of the samples. In Phase II, the toxicants were confirmed using a chemical analysis such as an ICP analysis. Finally, the toxicants were identified in phase III using a mass balance test (US Environmental Protection Agency 2007). Note, however, that Phases I and II were used to determine the main effects in this study. Five TIE treatments were designated: (1) pH adjustment to 3, 7, and 10 which reduce the toxicity by pH changes and cations, (2) pH adjustment/filtration (0.45 µm) which reduce the toxicity by solid materials, (3) pH adjustment/aeration which reduce the toxicity by volatile chemicals, (4) graduate pH which reduce the toxicity by ammonia, (5) EDTA chelation which reduce the toxicity of metal ion, finally the results was compared to baseline test which is control group. In Phase II, chemical measurement by ICP-OES and IC were conducted, and the results were then compared with the results of the ion exchange step. In the ion exchange step, a cation exchange resin (Amberite IR-120, Sigma Aldrich, USA) and anion exchange resin (Amberite IR 410, Sigma Aldrich, USA) were used to exchange ionic materials in the samples.

As the mine drainages contained a mixture of toxic metals, including As, Cd, Cu, Mn, and Zn, the cumulative criterion unit (CCU) was calculated as the sum of ratios between the stream metal concentrations and the metal criterion values for toxicity [Eq. (2)] (Hickey and Clements 1998; Clements et al. 2002). Here, the CCU is defined as

2 $${\text{CCU}} = \Sigma_{\text{i}} \left( {{\text{mi}}/{\text{ci}}} \right)$$

where *m*_*i*_ is the total recoverable metal concentration and *c*_*i*_ is the criterion value for the *i*th metal. Because the water hardness affects the toxicity and bioavailability of some metals, criterion values for Cd, Cu, Mn, and Zn were modified to account for variation in the water sample hardness. Hardness-adjusted LC_50_ values were then calculated using the correction equations that were derived from batch experiments using *Daphnia magna* (Table 1).

**Table 1:** Correction equations for hardness-adjusted toxicity values (LC_50_) of heavy metals (Cd, Cu, Mn, and Zn) results from 24 h acute toxicity test using *Daphnia magna*

Metals	Correction equation	
Cd	*LC*_*50*_ = 1.0035 log(*hardness*) − 1.5425	*r* ^*2*^ = 0.8257, *p* < 0.05
Cu	*LC*_*50*_ = 0.1988 log(*hardness*) − 0.3133	*r* ^*2*^ = 0.9126, *p* < 0.05
Mn	*LC*_*50*_ = 44.9173 log(*hardness*) − 56.9595	*r* ^*2*^ = 0.7968, *p* < 0.05
Zn	*LC*_*50*_ = 21.5845 log(*hardness*) − 34.1380	*r* ^*2*^ = 0.9349, *p* < 0.05

## Results and discussion

### Water characteristics of mine drainage

The water characteristics of mine drainage in the DS and ST mine with adjacent stream are summarized in Table 2. The samples commonly display acidic pH (<5), with leachate (DS-3) having the lowest pH (3.4). DS-2 showed a significant reduction of iron concentration in the effluent, whereas there was no significant difference in pH, EC, TDS, metal concentration rather than mine water (DS-1). The merged mine drainage (DS-4) demonstrated intermediate characteristics of DS-3 and DS-2. The adjacent stream (DS-5) shows a neutral pH (6.8), low conductivity (55 μS/cm) and low heavy metal concentration (<1 mg/L). The stream originates from a pond at upstream and it is estimated that the stream was not affected by the mining activities. The mixed mine drainage forms a mixing zone (DS-6) with the clean stream (DS-5); the mixed zone indicates that most factors are diluted by the mixing with clean water. In particular, the pH dramatically increased and the TDS, and sulfate and metal concentrations significantly decreased since the flow rate of the DS-5 is 300 times higher than that of mine drainage. The concentrations of metals in the downstream (DS-7) are quite similar to the results of the mixing zone, suggesting that the mixed mine drainage sufficiently reaches an equilibrium with the adjacent stream. In ST mine, a stream (ST-1) flows in front of the abandoned mining site, and two kinds of mine drainage are discharged into the adjacent stream. ST-1 shows that the leachate (ST-2) has acidic pH (4.5) and high sulfate concentration (150 mg/L). Due to the low concentration of heavy metals in ST-2, there is no significant effect shown in the first mixing zone (ST-3). The mine water (ST-4) represents the typical properties of mine drainage, with an acidic pH (3.0) and high concentration of sulfates and metals. In the second mixing zone (ST-5), the pH increased to 4.2 and most of the metal concentrations significantly decreased due to the dilution effect from the stream; consequently, the mine water negatively impacted the adjacent stream by lower pH and increasing the toxic metal concentration.

**Table 2:** Seasonal water characteristics of mine drainage in DS and ST mine

Sample		TU	PH	EC	Alkalinity	TDS	SO_4_^2−^	Fe^2+^	Tot-Fe	DO	Ca	Mg	Al	Mn	Cd	Cu	Zn
				(mS/cm)		(g/L)	(mg/L)										
DS-1	D	7.46	4.9	1.26	0.4	1.16	900	32.6	125	5.8	343	117	15.1	53.9	0.17	5.10	14.5
	R	1.94	4.5	2.02	n.d.^c^	1.01	2600	1.92	168	6.38	334	112	13.3	54.2	0.16	4.34	12.0
DS-2	D	8.64	3.9	1.18	n.d.	1.18	1400	0.96	8.06	3.8	389	121	14.3	55.9	0.08	1.88	9.80
	R	2.02	3.7	1.92	n.d.	0.96	2100	1.34	5.32	3.56	342	99.1	5.32	47.0	0.02	0.23	4.49
DS-3	D	55.9	3.4	1.00	n.d.	0.98	370	0.52	4.04	6.2	246	90.2	24.6	43.3	0.15	4.31	9.26
	R	21.7	3.2	1.42	n.d.	0.71	600	0.1	1.7	6.13	180	63.4	14.7	30.3	0.10	2.64	6.43
DS-4	D	14.5	3.9	1.11	n.d.	0.99	4	0.01	4.89	5.1	300	96.2	12.2	35.5	0.09	2.32	6.74
	R	13.9	3.8	1.33	n.d.	0.66	200	0.39	2.06	4.68	211	63.8	8.33	25.0	0.05	1.15	4.10
DS-5	D	–^b^	6.8	0.04	1.4	0.04	4	0.01	0.06	6.9	10.8	2.67	0.23	0.46	n.d.	0.03	0.11
	R	–	6.2	0.06	9.8	0.03	6	0.04	n.d.	6.48	7.3	5.07	0.03	0.05	n.d.	n.d.	n.d.
DS-6	D	1.20	6.9	0.06	1.2	0.05	17	n.d.	n.d.	7.5	11.3	3.02	n.d.	0.56	n.d.	0.01	0.12
	R	–	6.9	0.07	12.2	0.03	11	0.06	n.d.	6.86	6.92	5.07	0.05	0.10	n.d.	n.d.	n.d.
DS-7^a^	D	1.20	7.1	0.06	1.1	0.05	16	0.02	n.d.	7.9	11.3	2.9	n.d.	0.53	n.d.	0.01	0.11
ST-1	D	1.09	7.1	119	1.5	0.05	20	0.01	n.d.	5.05	14.7	2.37	0.09	n.d.	n.d.	n.d.	n.d.
	R	–	6.6	45.0	6.1	0.02	15	0.13	0.04	6.2	8.05	1.87	0.08	n.d.	n.d.	n.d.	n.d.
ST-2	D	1.10	4.5	443	n.d.	0.22	150	0.29	0.10	5.86	33.6	22.3	11.4	4.09	n.d.	n.d.	0.22
	R	2.17	5.0	78.0	n.d.	0.39	45	0.39	0.11	5.7	7.71	4.82	1.50	0.57	n.d.	n.d.	n.d.
ST-3	D	1.10	5.7	172	0.5	0.09	41	0.01	n.d.	4.73	18	5.55	0.12	0.58	n.d.	n.d.	n.d.
	R	–	6.7	49.0	8.5	0.05	10	0.04	n.d.	4.32	8.52	2.37	0.01	0.02	n.d.	n.d.	n.d.
ST-4	D	14.4	3.0	3690	n.d.	1.85	2300	3.90	32.5	5.47	220	277	243	49.8	n.d.	0.52	4.94
	R	9.98	3.3	1460	n.d.	0.73	800	0.80	9.7	5.37	140	93.1	75.1	15.4	n.d.	0.26	1.20
ST-5	D	1.90	4.2	608	n.d.	0.30	240	0.23	0.53	5.32	35.4	27.2	25.2	4.12	n.d.	n.d.	0.42
	R	1.32	4.1	393	n.d.	0.20	210	0.36	1.11	5.04	26.8	18.8	16.2	2.38	n.d.	n.d.	n.d.

*TU* average toxicity unit

^a^DS-7(R) was not measured the properties

^b^No effect

^c^Not detected

### WET results

**Fig. 2:**
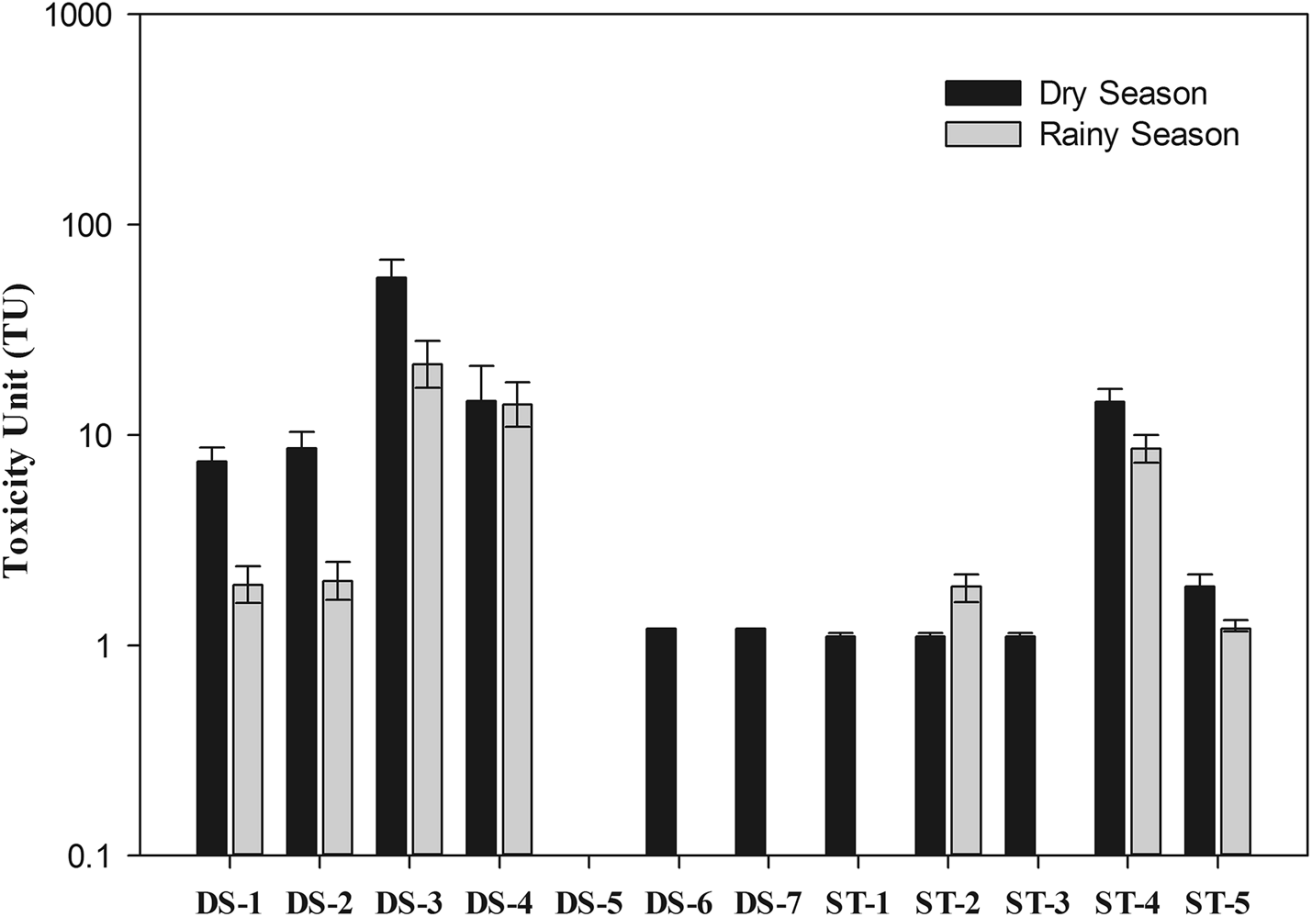
Seasonal toxic effect of mine drainage and adjacent stream (Dry Season: May 2012; Rainy Season: August 2012)

The WET results are illustrated in Fig. 2, which reveal that mine water (DS-1), treated water (DS-2), and leachate (DS-3) have a significant toxicity to *Daphnia magna*. Notably, DS-3 shows the highest toxicity (55.9 TU), which could originated from the strongly acidic pH of leachate (pH 3). Furthermore, the metal concentrations in leachate (DS-3) is lower than in the mine water (DS-1), including hardness ions such as calcium and magnesium, thus it might be considered that the toxic. DS-2 and DS-1 display a similar toxicity because the characteristics are also similar between these samples, i.e., there was no difference in pH, EC, TDS, metal concentration, and toxicity to *Daphnia magna*. The mixed mine drainage (DS-4) demonstrated the intermediated toxicity of mine water and leachate because of the mixing effect. The adjacent stream (DS-5) did not show any toxicity to *Daphnia magna* because there were little concentration of metal and neutral pH condition. In the mixing zone, the toxicity is dramatically decreased with increasing pH and decreasing TDS, sulfate and metal concentration. Toxic results also demonstrated the substantial reduction (14.5 TU to 1.2 TU) by the mixing effect with upstream water. Previous research have shown that the hydraulic dilution effect of contaminated water is important factor to assess the ecological effect in field study as a natural attenuation (Courtin-Nomade et al. 2012; Barber et al. 2013).

In the ST mine drainage, ST-1 showed low toxicity (1.0 TU), and the leachate (ST-2) shows low toxicity (1.1 TU) to *Daphnia magna* because of the low concentration of toxic metals in the leachate. ST-3 also demonstrated the low toxicity (1.0 TU). The mine water (ST-4) demonstrated a higher toxicity (14.4 TU) than the other samples in the ST mine. This toxicity could originate from the strong acidic pH (3.0) and high concentration of sulfates and metals, similar to that of the DS leachate (DS-3). In the second mixing zone (ST-5), the toxicity also dramatically decreased by the mixing effect (1.9 TU), though the toxicity in the stream remained hazardous to organisms in an aquatic environment. Consequently, the mine water negatively impacted the aquatic environment in terms of long term effect. In case of seasonal variation, the overall tendency of toxicity in the mine drainage and adjacent stream in this study is shown in Fig. 2, where the data shows that there was a notable decrease in the toxicity during rainy season. Generally, the toxicity of water in the rainy season is lower than in the dry season because of the dilution effect by rainfall.

### Toxicity characterization of DS mine drainage

**Fig. 3:**
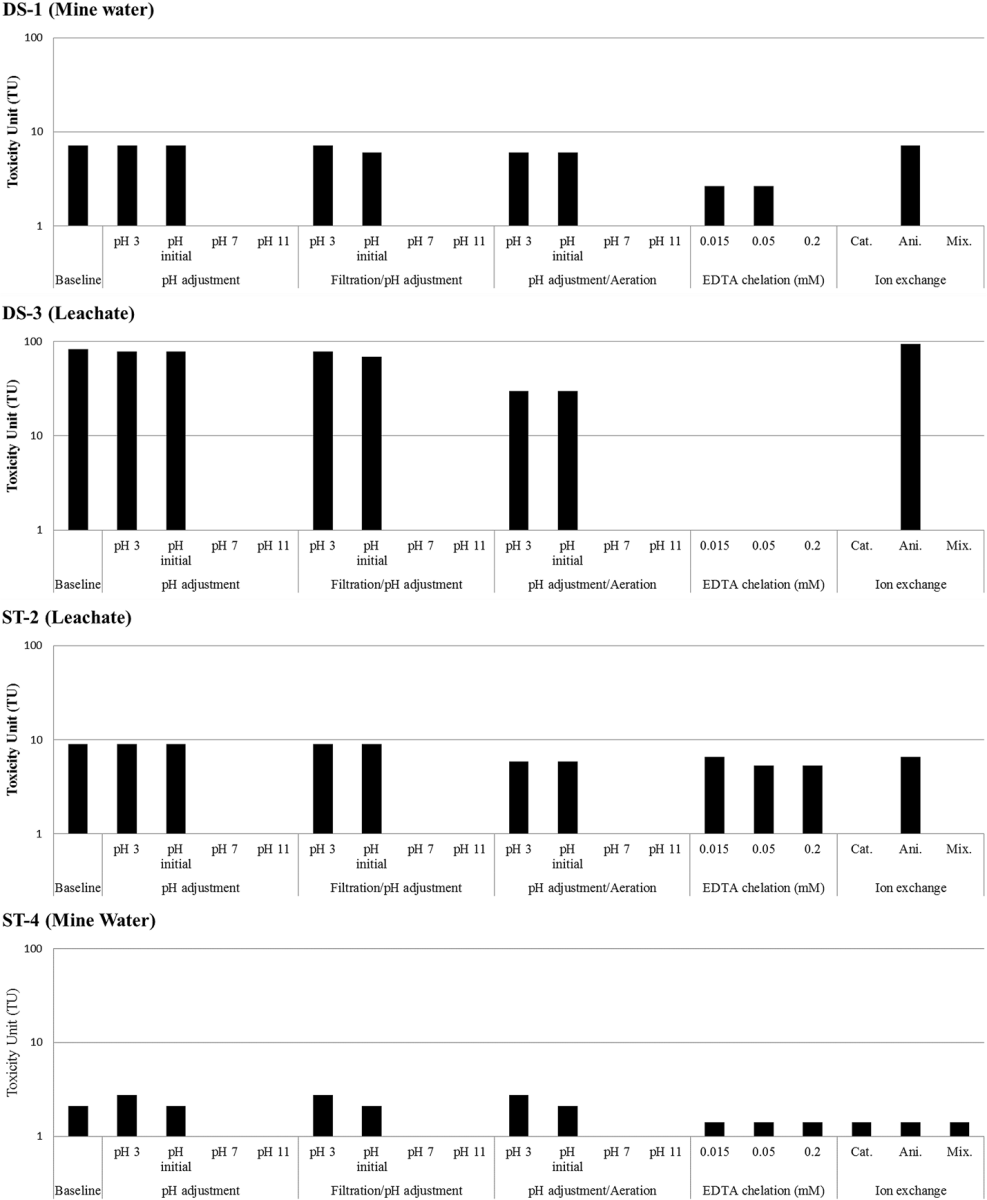
Toxicity identification evaluation of acid mine drainage (*DS-1* mine water in Dalsung mine, *DS-2* leachate in Dalsung mine, *ST-2* leachate in Samtan mine, *ST-4* mine water in Samtan mine)

The results of the toxicity identification evaluation are shown in Fig. 3. All baseline tests to verify the toxicity variation in the test are seen to be reasonable compared to the WET results as the difference in LC_50_ was below 2 %. In DS-1, the toxicity dramatically decreased after the pH adjustment to alkaline conditions, but there was little effect seen after manipulations of the filtration and aeration steps. Therefore, the main toxicant is not assumed to be the solid or volatile materials. Here, the toxicity decreased with the addition of the EDTA, implying that the heavy metals in the mine drainage can form metal chelate complexes which reduce the toxicity by non-available metal form (Hsieh et al. 2004). In the ion exchange steps, the samples filtered by the cation exchange and cation–anion combined resins displayed a significant reduction in the toxicity. Therefore cations could be a main toxicant in main drainage. Previous studies demonstrated that cationic metals such as cadmium, copper and lead were identified as a main toxicant by the step of cation exchange process in TIE procedure (Schiff et al. 2007; Li et al. 2011). Therefore, the main toxicant is posited to be a cationic material that contributes to *Daphnia magna* toxicity in this study. As shown in Table 2, Cd, Cu, and Mn are potential candidates for the main toxicant. Interestingly, the leachate had a 10 times higher toxicity than the mine water even though the concentration of heavy metals was lower in the leachate. This difference is likely due to the strongly acidic pH (pH 3.4), though the hardness of the leachate is also three times higher than DS-3. Previous research reported that a high concentration of hardness also can decrease the toxicity of *Daphnia manga* due to the competition of metal absorption between heavy metals and cations such as Ca and Mg (Paulauskis and Winner 1988; Di Toro et al. 2001), therefore, the hardness effect partially affects the toxicity of *Daphnia magna.*

### Toxicity characterization of ST mine drainage

In the ST samples, the concentration of toxic heavy metals is lower than in the DS mine; the TIE results showed that the pH is responsible for the toxicity variation, with the EDTA addition, cation exchange, and mixed bed exchange revealing the significant reduction of toxicity. As such, cations could be identified as the main toxicant in ST mine water. In case of the leachate, there were no significant differences with any manipulated treatments in the samples, as only a pH adjustment showed a reduction of toxicity. A chemical analysis revealed that the pH was similar to that of the DS mine, whereas the metal concentration of mine water was much lower than for the leachate in the DS mine (DS-3) (Table 2). However, the TU of the leachate in the DS mine is 10 times higher than that of ST-4 (mine water), even though there is a similar pH value. The data is compared in Table 3, indicating that the metal concentration (Cu, Mn and Zn) could be the main toxicant of AMD, though the pH adjustment results showed that the toxicity of *Daphnia magna* could change, even in the presence of metals. This variation is due the fact that metal could precipitate as a non-available form at a higher pH (Gadd and Griffiths 1977). Therefore, both the pH and metal concentration should be considered in order to estimate the impact of AMD.

**Table 3:** Chemical properties of manipulated each mine drainage by TIE procedures in DS and ST mine drainage (Dry season)

Sample		TU	Cd	Cu	Mn	Zn
			mg/L			
DS-1	Raw water	7.18	0.17	5.10	53.9	14.5
	Cation exchange	–^a^	–	0.02	0.11	0.02
DS-3	Raw water	83.3	0.15	4.31	43.3	9.26
	Cation exchange	–	–	0.01	0.07	0.01
ST-2	Raw water	2.09	–	–	4.09	0.22
	Cation exchange	1.41	–	–	–	–
ST-4	Raw water	8.93	–	0.52	49.8	4.94
	Cation exchange	1.47	–	–	0.01	–

^a^Not observed

### Toxicity evaluation results

To verify the effect of pH on *Daphnia manga*, a toxicity test was conducted, with the calculated LC_50_ being 4.35 ± 0.04. Previous research reported that the mortality of *Daphnia magna* could dramatically increase below pH 3.7 (Walton et al. 1982), and previous research (Alibone and Fair 1981; France 1982) already reported that acid pH (high concentration of hydrogen ion) directly affects the growth of *Daphnia magna.* In both metal mine and coal mine drainage, DS-3 and ST-4 demonstrated the extremely higher TU even though the metal concentration is lower than metal-rich mine drainage (DS-1). This is because the mine drainage is accompanied strongly acidic pH (pH < 3.3) with metal concentration. The acidic pH involves both the toxicity of hydrogen ions and metal activity, therefore the acid pH could be a key factor to determine the toxicity of mine drainage. Generally, the toxicity of metal mine drainage had a higher toxicity than coal mines because the toxic heavy metals entering the environment by acidic pH enhances the mobility of minerals (Gäbler 1997). However, the strongly acidic pH could be the variable factor even though there is similar concentration in mine drainage.

**Fig. 4:**
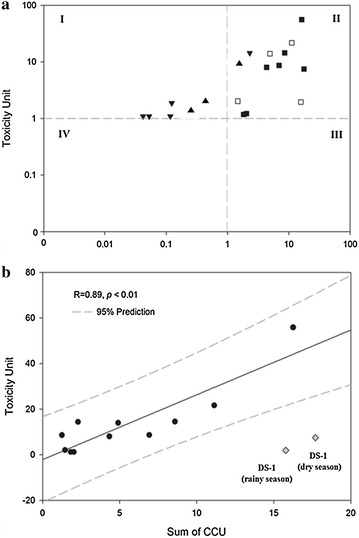
Comparison between cumulative criterion unit of toxic metals (As, Cd, Cu, Zn) and 24 h toxicity unit (**a** *filled square* DS, dry season, *unfilled square* DS, rainy season, *inverted filled triangle* ST, dry season, *filled triangle* ST, rainy season) and Linear regression analysis of CCU and TU (n = 11 in part II) (**b**)

Table 4 demonstrated the Comparison of 24 h acute toxicity (LC_50_) in *Daphnia magna* by As, Cd, Cu, Mn, and Zn with online database (ECOTOX database) which is summarized the toxicity of isolated metals by previously reported studies. The experimental LC_50_ data of each heavy metals to *Daphnia magna* demonstrated reasonable value in the range of the database, especially copper (Cu) revealed the highest toxicity (LC_50_; 0.13 mg/L). Furthermore, cadmium also demonstrated higher toxicity (LC_50_: 0.7 mg/L), whereas the LC_50_ value of manganese (43.2 mg/L) and zinc (14.1 mg/L) was relatively lower than that of copper and cadmium similarly the database. These results support the reason of higher toxicity of leachate in DS mine (DS-3). Chemical analysis data (Table 2) shows the concentration of copper extremely exceed the LC_50_ value, therefore the main toxicant could be copper in DS mine drainage. Although the copper concentration of mine water in DS mine (DS-1) is higher than DS-3, the reason of higher toxicity in DS-3 can be originated from the additional effect of acidic pH. The mine water in ST mine (ST-4) also similar pattern of the toxicity. ST-4 (highest TU) only contains copper concentration (Dry season: 0.52 mg/L; Rainy season: 0.26 mg/L) those exceed the LC_50_ value, and represent acidic pH (pH < 3.3). Even though the cooper and acidic pH can be dominant toxicant in mine drainage, other heavy metal effect cannot be negligible because the some metal concentration is close to LC_50_. In order to identify the effect of heavy metals and pH in mine drainage, cumulative criterion unit (CCU) and *Daphnia* toxicity were compared. The results in Fig. 4a indicate that most belong to part II, suggesting that the toxicity originates from the concentration of sum of toxic heavy metals (Cd, Cu, Mn and Zn). The results show a similar tendency in the TIE procedure, as the toxicity of the DS mine drainage can be affected by various heavy metals. In contrast, some samples from the ST mine drainage were in part I, i.e., the samples showed that toxicity occurred although there was a low concentration of heavy metals due to the effect of acidic pH to the mortality of *Daphnia magna*, and the main toxicant of ST mine could be acidic pH, however presence of heavy metals enhance the toxicity in ST mine drainage. In addition, a linear regression between the toxicity unit and sum of cumulative criterion unit was then estimated (Fig. 4b). In this regression, the statistical significance was reasonable (R = 0.89, p < 0.01), and most of the toxicity in samples could be correlated to the concentration of heavy metals as the samples were in the 95 % prediction range. Therefore the CCU model for estimating the negative effect of trace metal mixture was well explained to the negative effect of trace metals in acid mine drainage. However, the mine water from the DS mine drainage (DS-1) was not included in the significant range; the toxicity of the DS mine water was underestimated due to the change of water characteristics by the oxidation and precipitation due to the excessive iron concentration (during the sampling methodology. Therefore, actual toxicity of mine drainage was underestimated due to precipitation by the excessive iron concentration (>100 mg/L) present in the samples by the formation of amorphous iron precipitate and sorption process of heavy metals (Johnson 1986; Balintova and Petrilakova 2011).

**Table 4:** Comparison of 24 h Acute toxicity (LC_50_) in Daphnia magna by As, Cd, Cu, Mn, Zn with ECOTOX database

Elements	Experiment	ECOTOX^a^
	mg/L	
As	13.3	4.90–17.0
Cd	0.70	0.18–0.91
Cu	0.13	0.10–0.58
Mn	43.2	90.9
Zn	14.1	0.79–35.4

^a^ ECOTOX data base (2002) (http://cfpub.epa.gov/ecotox)

## Conclusion

Our study investigated the toxicity in mine drainage using WET and TIE conjunction procedures in conjunction with *Daphnia magna* for different types of acid mine drainage. The representative coal mine and metal mine were selected to identify the toxicity of mine drainage. WET data demonstrated that leachate in DS mine and mine water in ST mine is more toxic than other type of mine drainage due to the presence of cationic metals and strongly acidic pH. TIE data also revealed that the acidic pH and cationic metals such as cadmium, copper, manganese and zinc could be main toxicants in the DS mine, and the main toxicant of ST mine could be copper, manganese, and acidic pH. Among these metals, the LC_50_ value of copper was the lowest, and the concentration of copper exceed in DS-3 and ST-4 those represent the highest TU. Although the copper concentration of mine water in DS mine (DS-1) is higher than that of DS-3, the strongly acidic pH enhance the metal activity and bioavailability of metals. Most of mine drainage demonstrated positive correlation between CCU and TU, some of ST mine drainage demonstrated the single toxicity by acidic pH. The regression data between TU and sum of CCU demonstrated that the statistical significance was reasonable (R = 0.89; p < 0.01; 95 % prediction), however the excessive iron concentration in mine drainage cause the underestimating of toxicity due to the metal sorption onto the precipitate of amorphous iron.
